# Efficiency of non-invasive prenatal screening in pregnant women at advanced maternal age

**DOI:** 10.1186/s12884-021-03570-6

**Published:** 2021-01-26

**Authors:** Hui Zhu, Xiaoxiao Jin, Yuqing Xu, Weihua Zhang, Xiaodan Liu, Jinglei Jin, Yeqing Qian, Minyue Dong

**Affiliations:** 1 Jiaxing University Affiliated Women and Children Hospital, Jiaxing, Zhejiang, 314051 China; 2 Women’s Hospital, School of Medicine, Zhejiang University, Zhejiang, 310006 Hangzhou China

**Keywords:** Advanced maternal age (AMA), Trisomy, Non-invasive prenatal screening (NIPS), Fetal chromosomal aneuploidies, Prenatal screening method

## Abstract

**Background:**

Non-invasive prenatal screening (NIPS) is widely used as the alternative choice for pregnant women at high-risk of fetal aneuploidy. However, whether NIPS has a good detective efficiency for pregnant women at advanced maternal age (AMA) has not been fully studied especially in Chinese women.

**Methods:**

Twenty-nine thousand three hundred forty-three pregnant women at AMA with singleton pregnancy who received NIPS and followed-up were recruited. The sensitivity, specificity, positive predictive value (PPV), and negative predictive value (NPV), receiver operating characteristic (ROC) curves and the Youden Index for detecting fetal chromosomal aneuploidies were analyzed. The relationship between maternal age and common fetal chromosomal aneuploidy was observed.

**Results:**

The sensitivity, specificity, PPV, NPV of NIPS for detecting fetal trisomy 21 were 99.11, 99.96, 90.98, and 100%, respectively. These same parameters for detecting fetal trisomy 18 were 100, 99.94, 67.92, and 100%, respectively. Finally, these parameters for detecting trisomy 13 were 100, 99.96, 27.78, and 100%, respectively. The prevalence of fetal trisomy 21 increased exponentially with maternal age. The high-risk percentage incidence rate of fetal trisomy 21 was significantly higher in the pregnant women at 37 years old or above than that in pregnant women at 35 to 37 years old. (*Youden index* = 37).

**Conclusion:**

It is indicated that NIPS is an effective prenatal screening method for pregnant women at AMA.

## Background

With the implementation of the two-child policy in China, the number of pregnant women at advanced maternal age (AMA) has increased dramatically [1]. It has been shown that the pregnant women at AMA account for 33.4–46% in prenatal diagnosis centers [2–5]. The risk for fetal aneuploidies increases with maternal age. Invasive prenatal diagnosis should be recommended for women at AMA in some countries including China. Invasive prenatal diagnosis tests obtain the sampling of fetal genetic material through chorionic villus sampling (CVS) or amniocentesis. Although the both tests allow accurate diagnosis and have been implanted clinically for years, the invasive procedures still may result in miscarriage or intrauterine infection [6, 7]. Therefore, the invasive prenatal diagnosis is not accepted by some pregnant women. Non-invasive prenatal screening (NIPS) is the alternative for these women.

Cell-free fetal DNA-based NIPS has been proven to be of high sensitivity and specificity for detecting common chromosomal aneuploidies (trisomies 21, 18 and 13), with low false positive and false negative rates. Chen fang et al [8] recently reported that NIPS maintained high sensitivity (100,100, and 100%) and high specificity (99.89, 99.89 and 99.89%) for trisomies 21, 18 and 13, respectively. Moreover, clinical observations have indicated that NIPS has excellent performance in either high-risk or low-risk population of serological screening, and the detection efficiency is much higher than that of serological screening [9–12]. However, the application of NIPS in women at AMA is still rare, especially in Chinese population.

In this study, we intended to explore the clinical significance of NIPS to detect fetal trisomies 21, 18 and 13 in AMA pregnant women and to provide an appropriate prenatal screening program for these women.

## Methods

### Subjects

From February 1, 2015, to December 31, 2018, 29,343 AMA pregnant women (35 years old or older) who underwent NIPS and completed their pregnancy outcome follow-up at Jiaxing Maternal and Child Health Hospital and Women’s Hospital, Zhejiang University School of Medicine were recruited. The maternal age ranged from 35 to 55 years of age with a median of 37, and 151 cases were 45 years of age and over. The gestational age ranged from 12 to 30 weeks, and the median was 16 weeks. The inclusion criteria concluded: over 35 years old, singleton pregnancy, using NIPS as a screening test, and completing the follow-up investigation. Exclusion criteria follow the Chinese technical specification for prenatal screening and diagnosis of fetal free DNA in maternal blood [13] . The exclusion criteria included: less than 12 weeks of gestation, definite chromosomal abnormality in any of the couples, having received allogeneic blood transfusion or transplantation operation or allogeneic cell therapy within 1 year, fetal structural abnormalities, a family history of genetic diseases, malignant tumor, or other conditions that may affect the accuracy of NIPS. The gestational age was calculated according to their last menstruation period and verified by ultrasound in early pregnancy. The pregnancy outcomes were followed-up by checking the prenatal diagnosis database, delivery and infant record of hospital or registration system, or followed-up approximately 3 months after deliveries. The use of the data was approved with the Institutional Review Board [2016(lun)-21] and written informed consents were obtained from the all participants before NIPS. All the participants voluntarily chose NIPS or invasive prenatal diagnosis with full consents. Invasive prenatal diagnosis was provided for those at high risk of NIPS.

### NIPS

The venous blood of pregnant women was collected in tubes with EDTA at 4 °C. The plasma DNA was extracted and used for library construction. The fetal cell-free DNA fragments in the plasma were analyzed using the BGISEQ-100 sequencing platform. The risk for fetal trisomies 21, 18 and 13 were obtained through bioinformatics analysis [14, 15]. The normal range of the Z score was between − 3.0 and 3.0.

### Fetal karyotyping

Fetal karyotyping was recommended for women who were at high risk for trisomies 21, 18 and 13. The process of fetal karyotyping was described previously [16].

### Statistics

To evaluate the detective efficiency of NIPS in AMA women, the sensitivity, specificity, positive predictive value (PPV), negative predictive value (NPV) for detecting trisomies 21, 18 and 13 were calculated.

Receiver operating characteristic (ROC) curve was performed to investigate the significance for true positive percentage of fetal trisomy 21. Youden index was performed to identify the optimal cut-off point for true positive percentage of fetal trisomy 21 in different age groups, the sum of sensitivity and specificity [17]. Statistic analyses were performed using MedCalc version 19.0.4 (MedCalc Software Ltd., Ostend, Belgium).

## Results

### Results of NIPS

Twenty-nine thousand three hundred forty-three women at AMA who underwent NIPS and completed their pregnancy outcome follow-up were enrolled (Table 1). A total of 145 pregnant women were identified as being high-risk for fetal trisomy 21. Among them, 111 cases were confirmed to carry fetuses of trisomy 21. Eleven were false positive. Twenty-three cases did not receive diagnosis. Among those un-diagnosed, included was one case of spontaneous abortion due to premature rupture of membranes, three of stillbirth, five termination of pregnancies (TOP) due to fetal abnormalities (two cases of cardiac abnormalities, three cases of multiple malformations indicated), 13 of TOP without any diagnosis, and one case who refused prenatal diagnosis but delivered a baby of leukemia without karyotyping.

**Table 1:** Screening results of common fetal chromosomal aneuploidies

Trisomy	No. of cases with high-risk results	TP	FP	No. of cases without prenatal diagnosis	FN
T21	145	111	11	23	1
T18	70	36	17	17	0
T13	22	5	13	4	0

Data are presented as n (%), unless otherwise indicated. TP: true positive; FP: false positive; FN: false negative

A total of 70 pregnant women were identified to be at high risk for trisomy 18 (Table 1). Among them, 53 cases received diagnosis and 36 cases were confirmed to carry fetuses of trisomy 18. In addition, 17 cases did not receive prenatal diagnosis, including three cases of stillbirth, 11 TOP due to fetal malformations (eight of multiple malformations, three of cardiac abnormalities), and three TOP without any diagnosis.

Moreover, a total of 22 pregnant women were identified to be at high risk for trisomy 13 (Table 1). Among them, 18 cases received diagnosis and five were determined to carry fetuses of trisomy 13. Thirteen were false positive. Four cases did not receive the prenatal diagnosis and terminated their pregnancies, including three cases of multiple malformations, and one who refused diagnosis.

### Detective efficiency in AMA pregnant women

In order to estimate the detective efficiency of NIPS, we focused on the sensitivity, specificity, PPV, and NPV of trisomies 21, 18 and 13. As shown in Table 2, the sensitivity, specificity and NPV for NIPS for fetal trisomies 21, trisomy 18 and trisomy 13 were all over 99%. And, the PPV for fetal trisomies 21, trisomy 18 and trisomy 13 were 90.98, 67.92, and 27.78%, respectively.

**Table 2:** Detective efficiency of common chromosomal aneuploidies by NIPS

Trisomy	Sensitivity	Specificity	PPV (95% CI)	NPV (95% CI)	Rate of TP
T21	99.11 (94.62–99.99)	99.96 (99.93–99.98)	90.98 (84.08–95.19)	100 (99.98–100)	0.382
T18	100 (88.53–100)	99.94 (99.91–99.97)	67.92 (53.55–79.70)	100 (99.98–100)	0.123
T13	100 (51.09–100)	99.96 (99.92–99.98)	27.78 (12.17–51.20)	100 (99.98–100)	0.017

Data are presented as n (%), unless otherwise indicated. PPV, positive predictive value; NPV, negative predictive value; CI, confidence intervals

### The correlation between fetal trisomy 21 and maternal age

**Fig. 1:**
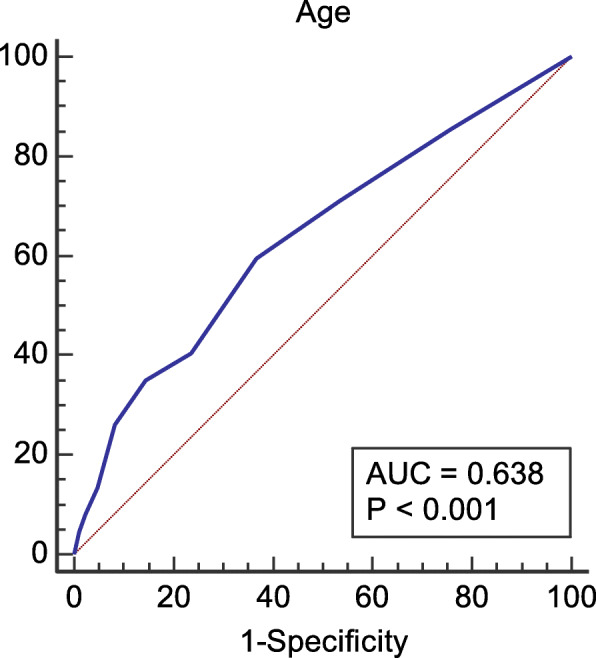
The AUC of true positive percentage of fetal trisomy 21 in AMA pregnant women. The area under the ROC curve of the fetal trisomy 21 in AMA pregnant women was 0.638 (*P* < 0.001)

**Fig. 2:**
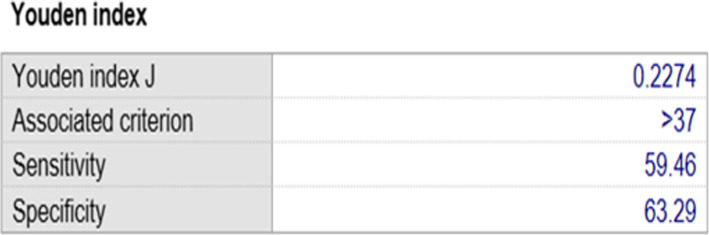
The Youden index of true positive percentage of fetal trisomy 21 in AMA pregnant women. The cut-off point was 37.0. The sensitivity and the specificity is 59.46 and 63.29%, respectively

As shown in Table 3, the rates of high-risk and true positive for fetal trisomy 21 were positively correlated exponentially with maternal age (*P* < 0.001). The area under the ROC curve of the fetal trisomy 21 in AMA pregnant women was 0.638 (Fig. 1). In addition, *Youden index* revealed the incidence of fetal trisomy 21 was significantly higher in the pregnant women at 37 years old and over than that in pregnant women at 35 to 37 years old (Fig. 2).

**Table 3:** High-risk rate and true positive percentage of fetal trisomy 21

Age(y)	No. of cases	No. of case with high-risk	Rate of cases with high-risk	TP	Rate of TP
35	7044	19	0.27	16	0.23
36	6557	26	0.40	16	0.24
37	4921	16	0.33	13	0.26
38	3824	28	0.73	21	0.55
39	2669	8	0.30	6	0.22
40	1863	12	0.64	10	0.54
41	1044	17	1.63	14	1.34
42	672	6	0.89	6	0.89
43	389	6	1.54	4	1.03
≥44	330	7	2.12	5	1.52

Data are presented as n (%), unless otherwise indicated. TP- true positive

## Discussion

In this study, we confirmed that NIPS had a high sensitivity, specificity and NPV for detecting fetal trisomies 21, 18 and 13 in AMA pregnant women. In addition, the incidence of fetal trisomy 21 increased with maternal age. These findings point to the clinical significance of NIPS to detect fetal trisomies 21, 18 and 13 in AMA pregnant women and may help doctors and pregnant women to choose a suitable prenatal screening and diagnosis way.

Serological screening is widely used. At present, as for the high-risk pregnant women, fetal karyotyping with amniotic fluid cells or cord blood cells is used as the diagnosis for fetal chromosomal abnormalities. In China, all AMA pregnant women are advised to undergo prenatal diagnosis. However, with the implementation of the two-child policy, the number of AMA pregnant women has increased significantly [1], which has greatly increased the demand for prenatal diagnosis [18]. However, amniotic fluid sampling or umbilical cord blood collection are invasive procedures, with the risk of miscarriage which was estimated at 0.5 to 1.0% [6, 19]. There is also a risk of infection in such procedures [20]. As a result, the overall utilization rate of both methods is low. Moreover, some pregnant women may have contraindications for invasive prenatal diagnosis, such as the high risk of inducing abortion, fever, increased tendency for bleeding, and infection [21]. Therefore, it is needed to find prenatal screening methods that better meet the clinical needs.

NIPS is a noninvasive prenatal screening technique for fetal aneuploidies. NIPS is based on high-throughput sequencing to detect cell-free fetal DNA (cffDNA) in maternal blood. In 1997, Lo et al. [22] found cffDNA in maternal blood and revealed that cffDNA was suitable for prenatal examination. However, it was not widely applied in clinic until the emergence of high-throughput sequencing [23]. Bianchi et al. [24] compared NIPS and serological screening in general population, which recruited 1914 women with singleton pregnancies from 21 centers in USA. Each sample was tested by both methods. The positive predictive values for NIPS and standard screening were 45.5 and 4.2% for trisomy 21, and 40.0 and 8.3% for trisomy 18, respectively. NIPS showed significantly better performances than serological screening. Meanwhile, Bianchi et al. [24] also found that the false negative rates were 0.3 and 0.2% for trisomies 21 and 18 as detected by NIPS, respectively, which were much lower than those of serological screening (3.6 and 0.6%, respectively). Similarly, in a study of 146,958 women [25], it was revealed that the sensitivity was 99.17, 98.24 and 100%, that the specificity was 99.95%,99.95 and 99.96%, that the PPV was 92.19, 76.61 and 32.84%, and that the NPV was 99.99, 100 and 100%, for trisomies 21,18 and 13, respectively. Using expanded non-invasive prenatal screening, Liang [26] demonstrated that the PPVs were 95, 82 and 46%, for trisomies 21, 18 and 13, respectively. Those findings obtained from large size of general populations were consistent with the results of ours, indicating NIPS has similar performance and is suitable for pregnant women at AMA.

Lots of investigations demonstrated that NIPS is superior to serological screening and suitable for the detection of trisomies 21, 18 and 13 in all high risk or low risk populations, AMA or not [24–26]. Thus, the International Society for Prenatal Diagnosis (ISPD), the American College of Obstetricians and Gynecologists (ACOG), the Royal College of Obstetricians and Gynecologists (RCOG), and the American College of Medical Genetics and Genomics (ACMG), have recommended NIPS as the preferred screening method for all pregnant women. Additionally, NIPS has been included in a national policy or national program in 14 European countries [27]. Considering the excellent efficiency of NIPS, NIPS could be promoted as the preferred screening method for AMA pregnant women. However, invasive screening methods such as amniotic fluid analysis and cord blood collection are still needed to carry out karyotype analysis for high-risk women identified by NIPS.

Since maternal age is closely associated with the incidence of fetal chromosomal abnormalities [28], we also studied the correlation between maternal age and the incidence of trisomy 21. Generally, the incidence increased with maternal age. This is consistent with previous report [29]. In the present study, among the 29,343 AMA pregnant women, 37 is the optimal cut-off point for identifying the fetal trisomy 21 with an AUC of 0.638 indicating that the prevalence of trisomy 21 was significantly higher in pregnant women aged 37 or older. Therefore, AMA pregnant women less than 37 years old can choose NIPS as a priority. As for those women aged 37 or older, invasive prenatal diagnosis should be advised first.

Although NIPS is confirmed as a test with a high sensitivity and specificity in common fetal aneuploidy, the false-positive results and false-negative results can still occur [30]. It was previously reported that NIPS had a false positive rate of 0.09, 0.13 and 0.13% for trisomies 21, 18 and 13, respectively [31]. Several factors might cause false-positive and false-negative results, like confined placental mosaicism (CPM) [32, 33], fetal mosaicism [32], vanishing twin [34, 35], maternal malignancy, low fetal concentration of low fetal fraction [36] and technical or human errors. In the present investigation, the false-positive rate were 0.04, 0.06, 0.04% for trisomy 21, trisomy 18 and trisomy 13, respectively. Additionally, the false-negative rate of trisomy 21, trisomy 18 and trisomy 13 were 0.89%, 0, 0, respectively. The false negative case in our study is a 39-year-old pregnant woman, whose NIPS results were low-risk at gestational age of 13 weeks. However, when she carried out regular prenatal organ screening at gestational age of 21 weeks, ultrasound revealed fetal edema and complete endocardial cushion defect. She was advised to accept invasive prenatal diagnosis and fetal trisomy 21 was confirmed. Therefore, there are still limitations in NIPS, which is a screening not a diagnostic method. Especially when the pregnant women meet any of the exclusion criteria, she should be advised to accept invasive prenatal diagnosis rather than NIPS.

We noted some shortcomings of this study. On the one hand, there were some cases without diagnosis in the high-risk population detected by NIPS. Many of these cases might have had fetal aneuploidies, especially those with ultrasound abnormalities or fetal death. Therefore, the PPV of fetal trisomies 21, 18, and 13 were likely to be higher than what were described here. On the other hand, the low incidence of trisomy 18 and trisomy 13 made it impossible to carry out an age stratification study as was done for the trisomy 21. Multicenter studies with larger sample sizes are expected in the future and that should provide additional data in support of optimizing prenatal screening and diagnosis strategies for AMA pregnant women.

## Conclusions

In summary, by analyzing the data from 29,343 AMA pregnant women, we demonstrated that NIPS is efficient for detecting fetal aneuploidies and is suitable for pregnant women at AMA.

## Data Availability

The data used or analyzed during the current study are included within the article. The datasets are not publicly available due to the hospital policy and personal privacy. However, the datasets are available from the corresponding author on reasonable request.

## Abbreviations

NIPS: Non-invasive prenatal screening
AMA: advanced maternal age
PPV: positive predictive value
NPV: negative predictive value
ROC: receiver operating characteristic
CVS: chorionic villus sampling
TOP: termination of pregnancies
cffDNA: cell-free fetal DNA
ISPD: International Society for Prenatal Diagnosis
ACOG: American College of Obstetricians and Gynecologists
RCOG: Royal College of Obstetricians and Gynecologists
ACMG: American College of Medical Genetics and Genomics
CPM: confined placental mosaicism
